# Differentiation of human adipose derived stem cells into Leydig‐like cells with molecular compounds

**DOI:** 10.1111/jcmm.14427

**Published:** 2019-07-10

**Authors:** Yong Chen, Chao Li, Weiping Ji, Long Wang, Xianwu Chen, Shenzhi Zhao, Zhangye Xu, Renshan Ge, Xiaoling Guo

**Affiliations:** ^1^ Center of Scientific Research The Second Affiliated Hospital and Yuying Children’s Hospital of Wenzhou Medical University Wenzhou PR China; ^2^ Department of Gastroenetrology The Second Affiliated Hospital and Yuying Children’s Hospital of Wenzhou Medical University Wenzhou PR China; ^3^ Department of Cardiology The Affiliated Hangzhou First People's Hospital of Zhejiang University School of Medicine Hangzhou PR China; ^4^ Department of Gynecology and Obstetrics The Second Affiliated Hospital and Yuying Children’s Hospital of Wenzhou Medical University Wenzhou PR China

**Keywords:** ADSCs, differentiation, EDS, LCs, testosterone, transplantation

## Abstract

Leydig cells (LCs) are the primary source of testosterone in the testis, and testosterone deficiency caused by LC functional degeneration can lead to male reproductive dysfunction. LC replacement transplantation is a very promising approach for this disease therapy. Here, we report that human adipose derived stem cells (ADSCs) can be differentiated into Leydig‐like cells using a novel differentiation method based on molecular compounds. The isolated human ADSCs expressed positive CD29, CD44, CD59 and CD105, negative CD34, CD45 and HLA‐DR using flow cytometry, and had the capacity of adipogenic and osteogenic differentiation. ADSCs derived Leydig‐like cells (ADSC‐LCs) acquired testosterone synthesis capabilities, and positively expressed LC lineage‐specific markers LHCGR, STAR, SCARB1, SF‐1, CYP11A1, CYP17A1, HSD3B1 and HSD17B3 as well as negatively expressed ADSC specific markers CD29, CD44, CD59 and CD105. When ADSC‐LCs labelled with lipophilic red dye (PKH26) were injected into rat testes which were selectively eliminated endogenous LCs using ethylene dimethanesulfonate (EDS, 75 mg/kg), the transplanted ADSC‐LCs could survive and function in the interstitium of testes, and accelerate the recovery of blood testosterone levels and testis weights. These results demonstrated that ADSCs could be differentiated into Leydig‐like cells by few defined molecular compounds, which might lay the foundation for further clinical application of ADSC‐LC transplantation therapy.

## 1 INTRODUCTION

Leydig cells (LCs) located at the testis interstitium can secrete about 95% of the body's testosterone, which is essential for the male phenotype development and spermatogenesis.^1^, ^2^, ^3^ Testosterone deficiency is characterized by sexual dysfunction, depressed mood, increased fatigue, decreased muscle mass and strength, changes in body composition and decreased cognitive function.^4^, ^5^, ^6^ A recent epidemiological study showed that near 4.5 million men in USA may suffer from testosterone deficiency.^5^, ^6^

To some extent, the testosterone deficiency can be relieved by exogenous testosterone replacement therapy.^7^, ^8^ However, this treatment disrupts the normal function of hypothalamic‐pituitary‐testicular axis, has no circadian rhythm and may cause a number of adverse reactions such as cardiovascular disorders, prostate tumorigenesis, et al.^9^, ^10^ Moreover, as physiological requirements for testosterone vary in individuals, the supplementation of exogenous testosterone is difficult to meet the demands of individual‐based treatment.^11^ LC transplantation had been demonstrated as a long‐acting and ideal physiological system of testosterone delivery.^12^ However, LC has the limited ability to proliferate, and account for only 2%‐4% of the total adult human testicular cells,^13^ limiting the clinical application of LC transplantation therapy.

Stem cells have significant pluripotency and self‐renewal characteristics, and had been universally used as seed cells in regenerative medicine or tissue engineering in recent years.^14^ Thus, the transplantation of stem cell‐derived LCs may be the alternative promising therapy for testosterone deficiency. Although several researches had attempted to induce stem cells, such as induced pluripotent stem cells (iPSCs),^15^ embryonic stem cells (ESCs),^16^, ^17^, ^18^ and mesenchymal stem cells (MSCs)^19^, ^20^ into steroidogenic cells using exogenous gene transcription factor transfection, it is not so safe for further clinical application. Human adipose derived stem cells (ADSCs) have been investigated as ideal seed cells because of the ease of obtaining, low immunogenicity and higher differentiation capacity.^21^ Currently, the differentiation of ADSCs into LCs using molecular compounds but not bringing in the exogenous genes had not been reported.

In our study, human ADSCs were induced into Leydig‐like cells (ADSC‐LCs) using a molecule‐compound‐based strategy. Transplantation of these ADSC‐LCs into the rat models treated with ethylene dimethanesulfonate (EDS)^22^ could promote the recovery of blood testosterone levels as well as reproductive organ weights. These findings will provide a new insight on the development of cell transplantation therapy for testosterone deficiency.

## 2 MATERIAL AND METHODS

### 2.1 Human adipose derived stem cell isolation and culture

The adipose tissues were obtained from the abdomen of five male donors with the mean age at 38 years by liposuction. The informed consent was acquired from every donor, and our research was approved by Human Research and Ethical Committee of Wenzhou Medical University. The fresh adipose tissues were microdissected into 1 mm^3^ pieces using a stereoscope, and then washed five times with PBS containing streptomycin (300 mg/mL) and penicillin (600 U/mL) to remove local anaesthetics, blood clots and red blood cells. The rest of tissues were digested using 0.1% type I collagenase at 37°C shaking table for 1 hour. Then these tissues were filtered through the cell strainer (100 μm) and centrifuged at 300 × g for 10 minutes. The deposit was suspended using low‐glucose Dulbecco's Modified Eagle's Medium (LG‐DMEM, Gibco) containing 10% fatal bovine serum (FBS, Gibco) and 1% penicillin/streptomycin (P/S, Gibco). The suspension was filtered through the nylon mesh (100 μm), and seeded into the 25 cm^2^ Petri dishes at a 37°C, 5% CO_2_ incubator. The medium was replaced with fresh medium every 3 days. When reaching 80%‐90% confluence, the cells would be passaged with 0.25% EDTA‐trypsin.

### 2.2 Adipogenic induction

ADSCs were conducted the adipogenic induction at passage 2 (P2) as the reported protocols.^21^ ADSCs (1 × 10^6^ cells/well) were seeded into six‐well cell culture dishes. The next day, cells were cultured under the adipogenic differentiation medium for 2 weeks, namely LG‐DMEM supplemented with 0.5 mM isobutyl methylxanthine (Sigma), 10 μM insulin (Sigma), 200 μM indomethacin (Sigma), 1 μM dexamethasone (Sigma), 10% FBS and 1% P/S. Cells were then fixed using 10% formaldehyde at room temperature for 30 minutes, rinsed three times with PBS, and darkly stained with 2% Oil Red‐O solution (Sigma) at room temperature for 10 minutes. Lastly, cells were washed three times with 70% alcohol, and were imaged under the inverted fluorescence microscope (OLYMPUS, Japan).

### 2.3 Osteogenic induction

ADSCs were conducted the osteogenic induction at passage 2 (P2) as the reported approach.^23^ ADSCs (1 × 10^6^ cells/well) were seeded into six‐well cell culture dishes. Then cells were cultured under the osteogenic differentiation medium for 2 weeks, namely LG‐DMEM supplemented with 1 μM dexamethasone (Sigma), 50 μM ascorbate‐2‐phosphate (Sigma), 100 μM glycerophosphate (Sigma), 10% FBS and 1% P/S. Then cells were rinsed three times with PBS, and were fixed by 4% paraformaldehyde at room temperature for 10 minutes. Then cells were rinsed three times using PBS, and stained using Alkaline Phosphatase Kit (Sigma). Lastly, cells were gently washed three times with deionized water, and were imaged under the inverted fluorescence microscope (OLYMPUS).

### 2.4 Human Leydig cell isolation and culture

LCs were acquired from five male donors with the mean age of 45 years by testes excision within 20 hours. Informed consent was obtained from every donor, and our research was approved by the Human Research and Ethical Committee of Wenzhou Medical University. These testes were used to isolate immature Leydig cells (ILCs), which can express all androgen synthases,^24^ and are capable of differentiation and proliferation.^25^ Briefly, the testes through testicular artery were perfused with M‐199 buffer (Gibco, NY, USA) containing DNase (0.25 mg/mL, Sigma) and collagenase (0.25 mg/mL, Sigma) for digestion about 15 minutes. Cell suspension was then filtered with nylon mesh (100 µm), and cells were separated using the Percoll Gradient (Sigma). The cells at the density of 1.070‐1.088 g/mL were harvested. The purity of ILCs was assessed by HSD3B1 immunohistochemical staining solution, which contained NAD^+^as a cofactor and 0.4 mM etiocholanolone (Sigma).^26^ The purity of ILCs was more than 95%.

The isolated ILCs at the density of 2×10^4^ cells/well were seeded into the 24‐well cell culture plates, and incubated at a 37°C, 5% CO_2_ incubator. The medium (LC‐medium) includes DMEM/F12 (Gibco), 2.5% horse serum (HS, Gibco), 5% FBS and 1% P/S. In order to gain adult LCs, the medium was changed into differentiation‐induced medium (DIM) with DMEM/F12, 5 ng/mL luteinizing hormone (LH, PeproTech, NJ, USA), 5 mM ITS (insulin, transferrin and selenium, Sigma) and 5 mM lithium chloride (Li, Sigma) as our team previous report.^27^

### 2.5 Animal

The Sprague Dawley (SD) rats (5 weeks old) were bought from the Laboratory Animal Center of Wenzhou Medical University, Wenzhou, China. They were raised under the environment with controlled temperature (23 ± 2°C), relative humidity of 45% to 55%, and a 12‐hour light/dark cycle. The standard diet and drinking water were accessed ad libitum. All experiments were approved by the Wenzhou Medical University's Animal Care and Use Committee, and were complied with the Guide for the Care and Use of Laboratory Animals.

### 2.6 Differentiation of human adipose derived stem cells into Leydig‐like cells

The point at which ADSCs (1×10^5^ cells/well) were seeded onto the six‐well culture plates in the ADSC medium was defined as day −5. From day −5 to 0, the medium was refreshed every 2 days. On day 0, ADSCs were changed in the differentiation‐inducing medium (ADSC‐DIM) containing DMEM/F12, 5 mM ITS, 5 ng/mL LH and1% BSA (Sigma). From 0‐7 days, 0.2 μM SAG (DHH agonist, Sigma), 5 μM 22R‐OHC (Steraloids, RI, USA) and 5 mM Li were added into ADSC‐DIM. From 7‐14 days, 5 ng/mL PDGF‐AA (Sigma), 5 ng/mL FGF2 (Sigma) and 10 μM Androgen (Sigma) were added into ADSC‐DIM. From 14‐18 days, 5 ng/mL LH (Sigma) and 0.2 μM SAG were added into ADSC‐DIM. From day 0 to 18, the medium was changed every 2 days by fresh ADSC‐DIM. On day 18, the cells were mechanically enriched through scraping away ADSC‐like cells (long spindle). From 18‐25 days, the remaining Leydig‐like cells were kept in Enrichment Medium contained DMEM/F12, 2.5% HS, 5% FBS, 1 × GlutaMAX (Invitrogen), 1 × sodiumpyruvate (Invitrogen) and 1% P/S for the subsequent assays. The medium was changed every 2 days by fresh Enrichment Medium.

### 2.7 Testosterone measurement by radioimmunoassay

The blood and cell culture supernatants were harvested for the quantitative detection of testosterone levels. For cell supernatant, 10 ng/mL LH was added into the medium (only DMEM/F12) in advance at least 3 hours to stimulate the testosterone production of LCs or ADSC‐LCs. The levels of testosterone were detected using a tritium based radioimmunoassay with the antibody of anti‐testosterone as previously reported.^28^ Standard samples containing testosterone ranging from 10 to 2000 pg/mL were prepared in triplicate. Standard or experimental samples were treated with antibody and tracer at 4°C overnight, and charcoal dextran suspension was used to separate the free and bound steroids. The bound steroid was mixed with the scintillation buffer and counted in the β scintillation counter (PE, CA, USA). The minimum detectable concentration of testosterone was 5 pg/mL. The inter‐assay and intra‐assay coefficient of variation was within 10%.

### 2.8 Immunofluorescence assay

Immunofluorescence was performed to identify ADSC‐LCs as previously reported.^23^ Cells were fixed by 4% paraformaldehyde (Sigma) for 15 minutes, and washed three times using PBS. Cells were then permeabilized using 0.1% TritonX‐100 at room temperature for 15 minutes, and incubated with 3% (w/v) BSA at room temperature for 1 hour. Cells were then incubated by primary antibodies (Table 1) overnight at 4°C, and then with FITC‐ or Cy3‐conjugated secondary antibodies (1:1000, Bioword, USA) at room temperature for 60 minutes. Lastly, cells were rinsed three times using PBS, and incubated with DAPI (Sigma) for nuclear staining for 15 minutes, and rinsed three times with PBS before examination under the inverted fluorescence microscope (OLYMPUS).

**Table 1:** Antibodies

Antibody	Species	Vendor (city, state, catalogue)	Dilution			
			WB	IC	FC	IF
β‐ACTIN	Rabbit	Cell Signaling Technology (Danvers, MA,12620)	1:5000	ND	ND	ND
STAR	Rabbit	Abcam (San Francisco, CA, ab133657)	1:1000	ND	ND	ND
SCARB1	Rabbit	Abcam (San Francisco, CA, ab217318)	1:1000	ND	ND	ND
SF‐1	Mouse	Santa Cruz (Santa Cruz, CA, sc‐393592)	1:500	ND	ND	ND
CYP11A1	Rabbit	Abcam (San Francisco, CA, ab75497)	1:1000	1:500	1:200	1:500
HSD3B1	Mouse	Abcam (San Francisco, CA, ab55268)	ND	ND	1:100	1:200
CYP17A1	Rabbit	Abcam (San Francisco, CA, ab125022)	ND	ND	1:200	1:500
HSD17B3	Rabbit	Abcam (San Francisco, CA, ab126228)	1:2000	ND	1:200	1:500
CD29	Mouse	Abcam (San Francisco, CA, ab27947)	1:1000	ND	1:100	1:500
CD44	Rabbit	Abcam (San Francisco, CA, ab119348)	ND	ND	1:100	ND
CD59	Mouse	Abcam (San Francisco, CA, ab9182)	1:1000	ND	1:100	ND
CD105	Mouse	Abcam (San Francisco, CA, ab2529)	ND	ND	1:100	ND
CD34	Rabbit	Abcam (San Francisco, CA, ab81289)	ND	ND	1:100	ND
CD45	Rabbit	Abcam (San Francisco, CA, ab10558)	ND	ND	1:100	ND
HLA‐DR	Rabbit	Abcam (San Francisco, CA, ab92511)	ND	ND	1:50	ND

Abbreviations: FC, flow cyt; IC, immunohistochemistry; IF, immunofluorescence; ND, not detected; WB, western blot.

### 2.9 Reverse transcription polymerase chain reaction (RT‐PCR) and real time polymerase chain reaction (qPCR)

Total RNAs from the samples were extracted using TRIZOL reagent (Gibco). The RNAs were reversely transcribed into cDNAs by the Reverse Transcription Kit (TOYOBO, Japan). The cDNAs were diluted 1:10, which were then used to conduct RT‐PCR or qPCR. RT‐PCR was conducted using the Authorized Thermal Cycler (Eppendorf, Hamburg, Germany). After amplification, 6 μL of each PCR product and 2 μL of loading buffer were mixed, and were electrophoresed into the 2% agarose containing nucleic acid dye (Sigma). Gel was scanned for further analysis. qPCR was conducted by the Thunderbird SYBR qPCR Mix (Takara, Tokyo, Japan) as the product instruction. Signals were harvested using the Light Cycler 480 Detection System (Roche, Basel, Switzerland). The relative gene expressions were normalized to GAPDH. The quantification was performed with the comparative 2^‐ΔΔCt^ approach. The sequences of primers were shown in Table 2.

**Table 2:** Primer information

Primer Symbol	Primer direction	Sequences (5’to 3’)	PCR (bp)	Accession
Lhcgr	Forward	CACATAACCACCATACCAGGAAA	124	NM_000233.3
	Reverse	AAGTCAGTGTCGTCCCATTGA		
Scarb1	Forward	GTCGCAGGCATTGGACAAAC	220	NM_001082959.1
	Reverse	CAGGACCTTGGCTCCGGATT		
Star	Forward	GGGAGTGGAACCCCAATGTC	78	NM_000349.2
	Reverse	CCAGCTCGTGAGTAATGAATGT		
Sf‐1	Forward	GGAGGCTTGCGAAGGAGAAG	105	NM_001178030.1
	Reverse	AGCTTACCCAACGGCGTG		
Cyp11a1	Forward	GCAGTGTCTCGGGACTTCG	102	NM_001099773.1
	Reverse	GGCAAAGCGGAACAGGTCA		
Hsd3b1	Forward	CACATGGCCCGCTCCATAC	90	NM_000862.2
	Reverse	GTGCCGCCGTTTTTCAGATTC		
Hsd11b1	Forward	AGCAGGAAAGCTCATGGGAG	131	NM_001206741.1
	Reverse	CCACGTAACTGAGGAAGTTGAC		
Cyp17a1	Forward	TATGGCCCCATCTATTCGGTT	161	NM_000102.3
	Reverse	GCGATACCCTTACGGTTGTTG		
Hsd17b3	Forward	GTCAACAATGTCGGAATGCTTC	91	NM_000197.1
	Reverse	TGATGTTACAATGGATGAGGCTC		
Dhcr7	Forward	AGGTGTGCGCAGGACTTTAG	174	NM_001360.2
	Reverse	TGGGAATGTTGGGTTGCGAT		
Igf1	Forward	AGAGCCTGCGCAATGGAATA	166	NM_012515.2
	Reverse	TTGGGTTGGAAGACTGCTGA		
CD29	Forward	CAAGAGAGCTGAAGACTATCCCA	137	NM_033668.2
	Reverse	TGAAGTCCGAAGTAATCCTCCT		
CD44	Forward	CTGCCGCTTTGCAGGTGTA	109	NM_001001392.1
	Reverse	CATTGTGGGCAAGGTGCTATT		
CD59	Forward	TTTTGATGCGTGTCTCATTACCA	106	NM_000611.5
	Reverse	ATTTTCCCTCAAGCGGGTTGT		
CD90	Forward	ATGAAGGTCCTCTACTTATCCGC	112	NM_001311160.1
	Reverse	GCACTGTGACGTTCTGGGA		
CD105	Forward	TGCACTTGGCCTACAATTCCA	107	NM_001114753.2
	Reverse	AGCTGCCCACTCAAGGATCT		
CD144	Forward	AAGCGTGAGTCGCAAGAATG	179	NM_001795.5
	Reverse	TCTCCAGGTTTTCGCCAGTG		
GADPH	Forward	ACAACTTTGGTATCGTGGAAGG	101	NM_001256799.2
	Reverse	GCCATCACGCCACAGTTTC		

### 2.10 Western blotting

Cells were rinsed using cold PBS, and were then lysed using lysis buffer containing protease inhibitor/1% phosphatase inhibitor mixture (Roche). The each sample with 50 μg of protein was applied into the 10% SDS‐PAGE, and was then transferred into the polyvinylidene difluoride membranes (PVDF, Sigma) using an electroblot apparatus. After blocked with the blocking solution (5% free‐fat milk) for 2 hours at 4°C, the PVDF membranes were then incubated using primary antibodies (Table 1) at 4°C overnight. The PVDF membranes were then rinsed five times (10 minutes each) using TBST, and incubated with HRP‐conjugated secondary antibody (1:3000, Bioword) at room temperature for 2 hours. The PVDF membranes were rinsed five times (10 minutes each) using TBS‐T. Bands were imaged by enhanced chemiluminescence (ECL, Pierce, USA).

### 2.11 Flow cytometry

Flow cytometry was conducted as the reported method.^21^ Briefly, cell samples were fixed using 4% paraformaldehyde in PBS, and permeabilized using 0.1% TritonX‐100 (Sigma). Cells were then labelled with primary or isotype control antibodies at 4°C for 30 minutes. Primary or isotype control antibodies were labelled with fluorophore conjugated secondary antibody at 4°C for 30 minutes. The labelled samples were detected by flow cytometry analyser (BD, USA).

### 2.12 Tagging ADSC derived Leydig‐like cells (ADSC‐LCs) with PKH26

The standard protocol was performed according to PKH26 Product Information Sheet (Sigma, MINI2). Briefly, the cell suspension with 2×10^7^ cells was centrifuged (400 × g, 5 minutes), and then were washed once using fresh LG‐DMEM without FBS. After centrifugation, the supernatant was removed, and 1 mL of Diluent C was added. Cells were resuspended with gentle pipetting to ensure complete dispersion. 2 × Dye Solution (4 × 10^‐6^ M) was prepared through adding 4 μL of PKH26 ethanolic dye solution into 1 mL of Diluent C, and mixed them well. Then, 1 mL of 2 × Dye Solution was rapidly added into the cell suspension. Final concentration was 2 × 10^‐6^ M PKH26 for 1×10^7^ cells/well. The mixing suspension was incubated using periodic mixing at room temperature for 5 minutes. The staining was stopped through adding 2 mL of FBS. Then the suspension was centrifuged (400 × g, 10 minutes) and rinsed three times. Finally, the cells tagged with PKH26 were transfer to fresh wells and used for transplantation.

### 2.13 Transplantation of ADSC derived Leydig‐like cells (ADSC‐LCs) in vivo

For assessment whether ADSC‐LCs could facilitate the recovery of testosterone deficiency of rats, ADSC‐LC transplantation was conducted according to the previous report with some modifications.^29^ Sixty 49‐day‐old male SD rats (n = 5 for each group at each time point) were used in this study. Water containing 210 mg/L Cyclosporin A (Sigma) was given to these rats throughout the experiment to prevent allograft rejection. Before cell transplantation, male rats were performed a single intraperitoneal injection of EDS (75 mg/kg, Pterosaur Biotech Co., Ltd., Hangzhou, China). EDS treatment would eliminate LCs in the adult testes of rats.^30^ Then, ADSC‐LCs labelled with PKH26 (red) were resuspended manually in a 15 mL tube. Cells were then washed twice using PBS and centrifuged (200 × g, 5 minutes). Lastly, each cell pellets were resuspended in PBS for transplantation. Cells were loaded into a 1 mL syringe for injection into the testis of adult male SD rats with EDS treatment. Approximately 2×10^6^ PKH26‐labelled ADSC‐LCs were injected into the parenchyma of rat testes. The control rats with EDS treatment received a testicular injection of PBS. Testes from all rats were examined at 0, 7, 14 and 21 days after EDS treatment.

### 2.14 Immunohistochemistry

One testis from each rat was used for immunohistochemistry. The rats were killed using the overdose of sodium pentobarbital (Sigma). Testes were removed, and were fixed with 4% paraformaldehyde at 4°C overnight. Then testes were dehydrated using a graded series of xylene and ethanol, and were then embedded into paraffin. Five‐micrometer‐thick sections were cut, de‐waxed in water and then were mounted on the glass slides. Antigen retrieval was conducted with the microwave irradiation in 10 mM citrate buffer (pH 6.0) for 10 minutes, after which endogenous peroxidases were blocked for 30 minutes by 0.5% of H_2_O_2_ in methanol. Some sections were then fixed using 4% paraformaldehyde for 15 minutes and rinsed three times using PBS. Then they were permeabilized using 0.1% TritonX‐100 in PBS for 15 minutes at room temperature, and were then incubated with 3% (w/v) BSA for 1 hour at room temperature. These sections were then incubated with CYP11A1 polyclonal antibody for 2 hours at room temperature, and then with FITC‐conjugated secondary antibodies for 1 hour at room temperature. These sections were rinsed three times using PBS. Then, the sections were incubated with DAPI (10 μg/mL, Sigma) for nuclear staining for 15 minutes, and rinsed three times using PBS. The sections were cover‐slipped with resin (Thermo Fisher Scientific, Waltham, UK). Lastly, they were examined under the inverted fluorescence microscope (OLYMPUS). Cells with CYP11A1 positive staining in the interstitial area represent LCs.^31^

Other sections were directly incubated with CYP11A1 polyclonal antibody diluted 1:1000 for 2 hours at room temperature. Diaminobenzidine was used for visualizing antibody‐antigen complexes, positive labelling LCs by brown cytoplasmic staining. Mayer haematoxylin was applied in counterstaining. The sections were then dehydrated in graded concentrations of alcohol and cover‐slipped with resin (Thermo Fisher Scientific, Waltham, UK). Lastly, they were examined by a fluorescence microscope (LEICA).

### 2.15 Enumeration of Leydig cell number by stereology

To enumerate CYP11A1 positive LC numbers, sampling of the testis was performed according to a fractionator method as our previous report.^32^ Identification of all LC lineages was done by the staining of CYP11A1. About 10 testis sections per rat were sampled from each testis. The total number of LCs was calculated by multiplying the number of LCs counted in a known fraction of the testis by the inverse of the sampling probability.

### 2.16 Statistical analysis

All experiments were performed at least thrice, and the data are presented as the mean ± SEM. Statistical analyses were evaluated using an unpaired Student's *t* test or one‐way ANOVA for more than two groups. *P* < 0.05 was considered statistically significant.

## 3 RESULTS

### 3.1 The isolation and identification of human adipose derived stem cells

**Figure 1:**
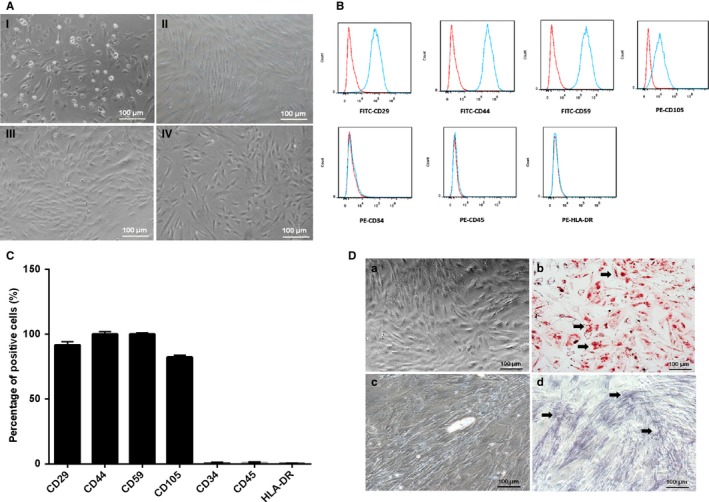
The isolation and identification of human adipose derived stem cells (ADSCs). A, The isolation and culture of ADSCs. (I) The primary of ADSCs; (II) The passage 1 of ADSCs; (III) The passage 2 of human ADSCs; (IV) The passage 3 of human ADSCs. B, Flow cytometry analysis of surface phenotypes of ADSCs. C, The histogram of cell surface antigens detected by flow cytometry. D, Adipogenic and osteogenic differentiation of ADSCs in vitro. (b) Oil red‐O staining in ADSCs was positive after cultured in the medium of adipogenic induction (black arrow); (d) Alkaline phosphatase staining in ADSCs was positive after cultured in the medium of osteogenic induction (black arrow); (a and c) negative control

Human ADSCs could be isolated from liposuction adipose tissue using type I collagenase digestion. Generally, after isolation for 6 days, blood cells could be removed through changing medium every 2 days, whereas primary ADSCs could attach to the walls well, and the morphology looked like a long‐spindle (Figure 1A I). They could continue to grow and proliferate. When reaching 80%‐90% confluence, they would be passaged using 0.25% EDTA‐trypsin, respectively passage 1 (P1) (Figure 1A II), passage 2 (P2) (Figure 1A III) and passage 3 (P3) (Figure 1A IV).

The surface antigens of ADSCs at P1 were assayed through flow cytometry. The results showed that the isolated ADSCs could positively express CD29, CD44, CD59 and CD105, but negatively express CD34, CD45 and HLA‐DR (Figure 1B and 1C), which were almost consistent with the reported ADSC identification.^33^ To demonstrate the pluripotency of ADSCs, the induction of adipogenesis and osteogenesis differentiation in ADSCs were conducted. The differentiated ADSCs were positively stained by Oil red‐O solution, which showed the lipid droplets formed on the cytoplasm of cells (Figure 1Db). The specific indicators of osteogenic differentiation were the secretion of ECM, which could be stained by alkaline phosphatase. The results showed that the differentiated ADSCs could be positively stained with alkaline phosphatase (Figure 1Dd). These results confirmed that cells derived from human adipose tissues were adipose derived stem cells (ADSCs).

### 3.2 Differentiation of human adipose derived stem cells into Leydig‐like cells (ADSC‐LCs)

**Figure 2:**
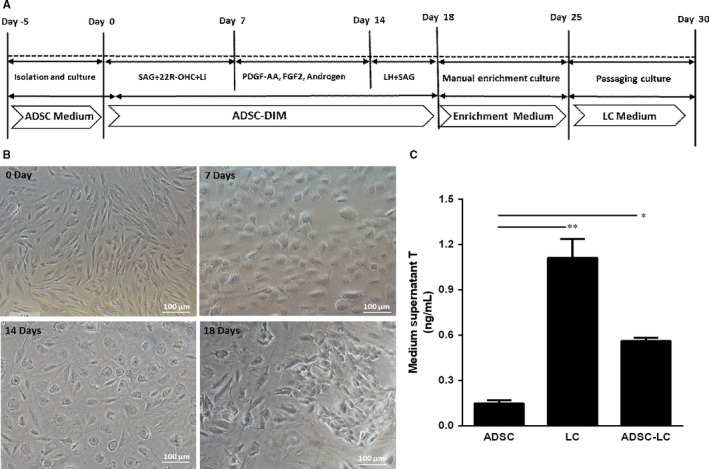
Differentiation induced pluripotent stem cells (ADSCs) into Leydig‐like cells (ADSC‐LCs) based on molecular compounds. A, The schematic illustration of differentiation protocol of ADSC‐LCs. B, Morphological changes in the differentiation of ADSC‐LCs under inverted microscope on day 25. C, Medium testosterone (T) levels in different groups by radioimmunoassay. Mean ± SE, n = 5. *P < 0.05, **P < 0.01 designate significant differences

From day −5 to day 0, the passaged ADSCs (P1) were cultured in the ADSC medium, which was refreshed every 2 days. When the cells reached about 100% confluence on day 0, the medium of ADSCs was switched into the differentiation‐inducing medium (ADSC‐DIM) with the sequential supplement by defined molecular compounds at different time points for 18 days in order to promote the proliferation and differentiation of ADSCs. When the partial ADSCs were successfully differentiated into Leydig‐like cells (ADSC‐LCs) on day 18, these cells would be manually enriched by scraping away ADSC‐like (long‐spindle) cells, and they were cultured in the Enrichment Medium for a week. Furthermore, they were passaged and cultured in LC Medium for the following experiments. The schematic illustration is showed in Figure 2A. ADSCs grew well and reached about 100% confluence on day 0. After differentiation for 7 days, the shape of cells became oval from long‐spindle with strong stereoscopic impression, but on day 14, the stereoscopic impression of these oval cells was disappeared, and on day 18, the partial Leydig‐like cells (ADSC‐LCs) exhibited ellipse shapes. These cells were tended to grow together to form clusters (Figure 2B). Under the stimulus with 10 ng/mL LH for 3 hours, the enrichment ADSC‐LCs could secrete testosterone (T) into the medium, and the level of T was more than that of ADSCs, but less than that of LCs (Figure 2C). These results suggested that our approach based on the molecular compound induction is able to differentiate the partial ADSCs into Leydig‐like cells.

### 3.3 Identification of Leydig‐like cells derived from human adipose derived stem cells (ADSC ‐LCs)

**Figure 3:**
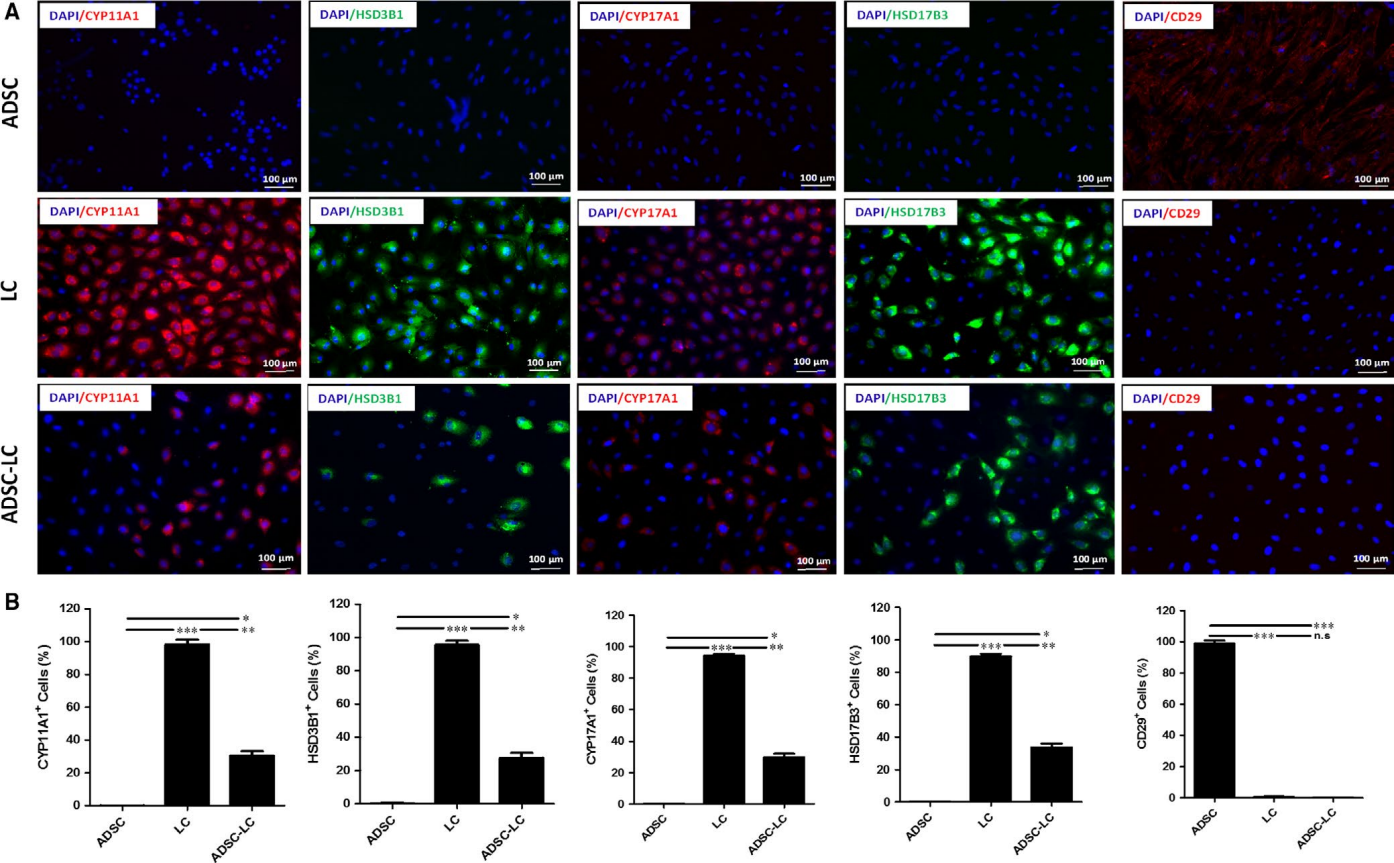
Identification of Leydig‐like cells derived from induced pluripotent stem cells (ADSC‐LCs). A, The detection of protein biomark expressions of Leydig cells or ADSCs using immunofluorescence assays in ADSCs, LCs and ADSC‐LCs. B, The statistical analysis of immunofluorescence. Mean ± SE, n = 5. *P < 0.05, **P < 0.01, ***P < 0.001 designate significant differences. n.s >0.05 designates no significant difference

After differentiation and enrichment, the immunofluorescence assay was used to identify the expressions of LC or ADSC protein biomarkers in ADSC‐LCs. The results showed that ADSC‐LCs could partially positively express LC biomarkers such as CYP11A1, HSD3B1, CYP17A1 and HSD17B3, but negatively express ADSC biomarkers CD29. The undifferentiated ADSCs negatively express CYP11A1, HSD3B1, CYP17A1 and HSD17B3, but positively express CD29. LCs strongly express CYP11A1, HSD3B1, CYP17A1 and HSD17B3, but negatively express CD29 (Figure 3A). The statistical data on the positive percentages of biomarker expressions were shown in Figure 3B. The results showed that the percentages of positive cells expressing LC markers such as CYP11A1, HSD3B1, CYP17A1 and HSD17B3 in ADSCs were 0.12%, 0.35%, 0.23% and 0.43%, respectively, which were lower than these of LCs (98.28%, 95.53%, 94.26%, 89.74%) and ADSC‐LCs (30.65%, 27.71%, 29.81%, 33.81%). In addition, the percentages of positive cells expressing ADSC marker CD29 in ADSCs were 98.81%, which were higher than those of LCs (0.67%) and ADSC‐LCs (0.34%).

These results demonstrated that the partial ADSCs were successfully differentiated into Leydig‐like cells based on the molecular compound induction.

### 3.4 Identification of Leydig‐like cells derived from human adipose derived stem cells (ADSC ‐LCs)

**Figure 4:**
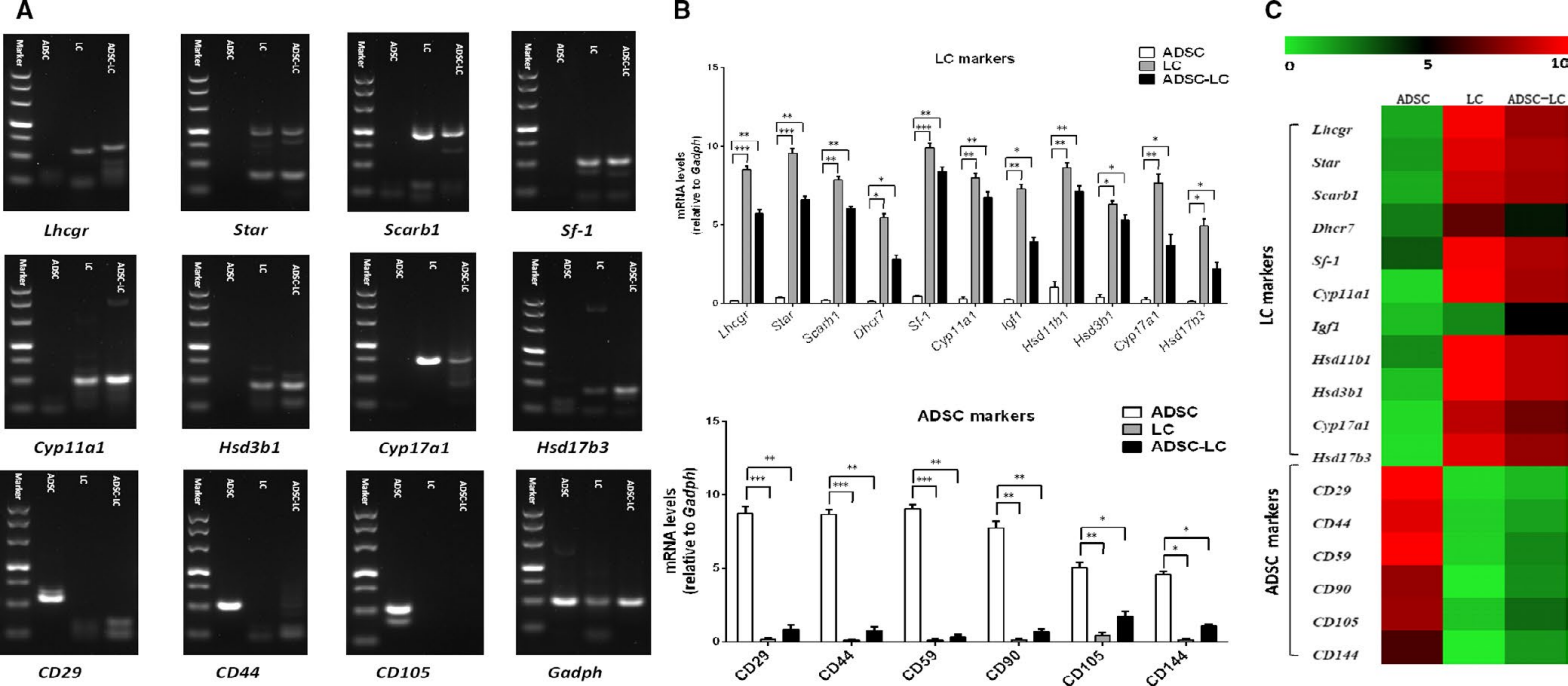
Identification of Leydig‐like cells derived from induced pluripotent stem cells (ADSC‐LCs) by gene expression assays. A, The detection of expressions of Leydig cell or ADSC relative genes using reverse transcription polymerase chain reaction (RT‐PCR) in ADSCs, LCs and ADSC‐LCs. B, The comparation of expression levels of Leydig cell or ADSC relative genes using qPCR in ADSCs, LCs and ADSC‐LCs. C, The heat map of qPCR results in ADSCs, LCs and ADSC‐LCs (green means low expression level and red represents high expression levels). Mean ± SE, n = 5. *P < 0.05, **P < 0.01, ***P < 0.001 designate significant differences

RT‐PCR assay was also conducted to characterize the expressions of LC or ADSC gene biomarkers in ADSC‐LCs. The results displayed that ADSC‐LCs could positively express LC gene biomarkers such as *Lhcgr*, *Star*, *Scarb1*, *Sf‐1*, *Cyp11a1*, *Hsd3b1*, *Cyp17a1* and *Hsd17b3*, which were necessary for the testosterone synthesis, but negatively express ADSC gene biomarkers such as *CD29*, *CD44* and *CD105*, that was similar to LCs but was opposite to ADSCs (Figure 4A). Meanwhile, qPCR assay was performed to compare the levels of LC or ADSC related gene expressions among them. The data showed that the levels of LC related genes such as *Lhcgr*, *Star*, *Scarb1*, *Dhcr7*, *Sf‐1*, *Cyp11a1*, *Igf1*, *HSD11b1*, *Hsd3b1*, *Cyp17a1* and *Hsd17b3* in ADSC‐LCs were significantly higher than those of ADSCs but lower than those of LCs. In addition, the levels of ADSC related genes including *CD29*, *CD44*, *CD59*, *CD90*, *CD105* and *CD144* in ADSC‐LCs and LCs were very low, which were significantly less than those of ADSCs (Figure 4B). The heat map was quantified to more visually exhibit the consequences of qPCR. Red means the gene level is high, and green means the gene expression level is low (Figure 4C). These results also demonstrated that ADSCs could be differentiated into Leydig‐like cells based on the induction method of molecular compounds.

### 3.5 Identification of Leydig‐like cells derived from human adipose derived stem cells (ADSC‐LCs)

**Figure 5:**
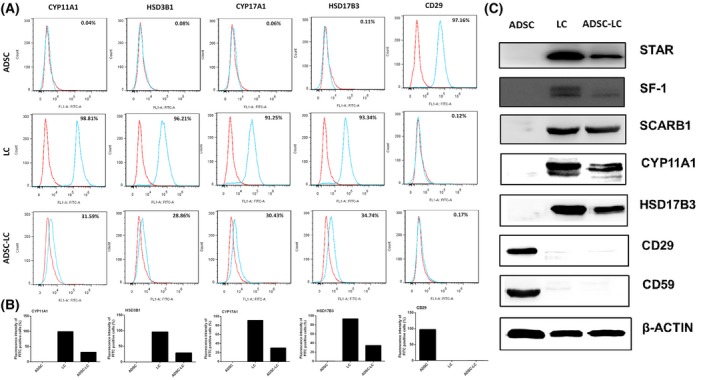
Identification of Leydig‐like cells derived from induced pluripotent stem cells (ADSC‐LCs). A, Representative flow cytometry histograms for CYP17A1, HSD3B1, HSD11B1 and OCT4 in ADSCs, LCs and ADSC‐LCs. B, The quantitative histogram of flow cytometry assays. C, The measurement of biomark protein expressions of Leydig cells or ADSCs using Western blotting in ADSCs, LCs and ADSC‐LCs

The flow cytometry histograms were employed to assess the population levels of LC biomarkers CYP11A1, HSD3B1, CYP17A1, HSD17B3 and ADSC biomarker CD29 in ADSCs, LCs and ADSC‐LCs. ADSC‐LCs could express LC‐like populations with CYP11A1 (31.59%), HSD3B1 (28.86%), CYP17A1 (30.43%) and HSD17B3 (34.74%), while they expressed ADSC populations with CD29 (0.17%). These properties were similar to those in LCs, which expressed CYP11A1 (98.81%), HSD3B1 (96.21%), CYP17A1 (91.25%), HSD17B3 (93.34%) and CD29 (0.12%), but were different to those in ADSCs, which expressed CYP11A1 (0.04%), HSD3B1 (0.08%), CYP17A1 (0.06%), HSD17B3 (0.11%) and CD29 (97.16%) (Figure 5A and 5B).

Western blotting assay was also used to identify the expressions of LC or ADSC protein biomarkers in ADSC‐LCs. The results showed that ADSC‐LCs could positively express LC biomarkers such as STAR, SCARB1, SF‐1, CYP11A1 and HSD17B3, which were androgen biosynthetic enzymes for testosterone synthesis, but negatively express ADSC biomarkers CD29 and CD59. These protein expressions in undifferentiated ADSCs were contrary to ADSC‐LCs, and LCs were in consistent with ADSC‐LCs (Figure 5C).

Taken together, these results further suggested that ADSCs could be partially differentiated into Leydig‐like cells based on the induction method of molecular compounds.

### 3.6 Transplantation of Leydig‐like cells derived from human adipose derived stem cells (ADSC ‐LCs) into the testes of rats with EDS treatment

**Figure 6:**
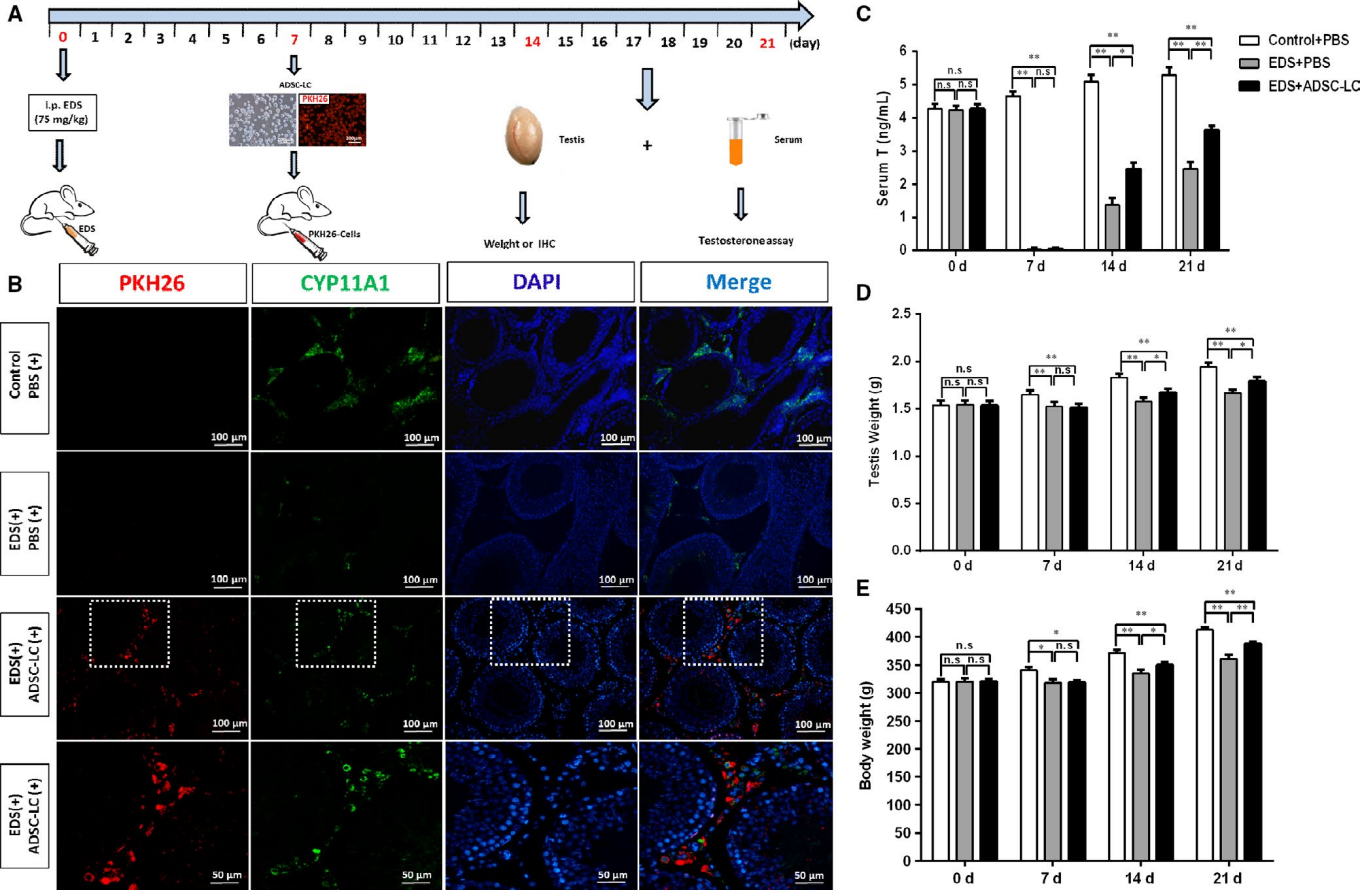
Transplantation of Leydig‐like cells derived from induced pluripotent stem cells (ADSC‐LCs) into the testes of rats with ethylene dimethanesulfonate (EDS) treatment. A, The schematic illustration of experimental procedure used for cell transplantation. B, Immunofluorescent staining showed the accumulation of cells were positive for PKH26 (red) and CYP11A1 (green) in the interstitial area of the testis at 2 wk after grafting. Nuclei were stained with DAPI (blue). The bottom panels showed higher‐magnification images of the dotted boxes in the lower‐magnification images. Control/PBS(+), rats without EDS treatment received PBS injection 7 d later; EDS(+)/PBS(+), EDS‐treated rats receiving PBS injection 7 d later; EDS(+)/ADSC‐LCs(+), EDS‐treated mice receiving ADSC‐LC transplantation 7 d later. C, Blood testosterone (T) levels at different time points by radioimmunoassay. D, The testes weights were assayed at different time points. E, The bodyweights of the testes were assayed at different time points. Mean ± SE, n = 5. *P < 0.05, **P < 0.01 designate significant differences

To assess whether ADSC‐LCs have the ability to survive and function in vivo, these cells were transplanted into the parenchyma at the cranial pole of the rat testes with EDS treatment on day 7. A single injection of EDS (75 mg/kg) in adult rat could eliminate the LCs in the testes to cause a dramatic decline in the levels of blood testosterone.^31^, ^34^ ADSC‐LCs stained with PKH26 (a red fluorescent dye) were injected into the recipient rats. On day 0, 7, 14 and 21 after EDS treatment, the blood and testes were harvested for analyses (Figure 6A). After 14 days of cell transplantation, the PKH26‐labelled ADSC‐LCs (red) were exclusively distributed in the interstitium of the testis, and expressed the LC‐specific marker CYP11A1 (green). In EDS‐treated rats with PBS administration, the CYP11A1‐positive cells were very less in the interstitium, but PBS injected rats without EDS treatment (control) strongly expressed CYP11A1 (Figure 6B). Furthermore, after EDS administration, the levels of blood testosterone were dramatically declined to the undetectable bottom on day 7 and recovered gradually, suggesting that EDS could specifically eliminate the testosterone‐producing LCs in the adult rats. In the EDS‐treated rats, the levels of blood testosterone began to increase on day 14, which was recovered to about 20% of control rats. Notably, the levels of blood testosterone in EDS treated rats with ADSC‐LC transplantation were higher than that of EDS treated rats with PBS injection, but were even lower than that of control rats with PBS injection on day 14 and 21 (Figure 6C). Testosterone plays an important role in maintaining the normal weights of reproductive organs.^35^ After exposure to EDS, the weights of testes were dramatically reduced in the EDS‐treated rats on day 7, but this decrease could be rescued by ADSC‐LC transplantation. Quantitative results showed that the absolute weights of the testes in EDS treated rats with ADSC‐LC transplantation were higher than that of EDS treated rats with PBS injection, but both were lower than that of control rats with PBS injection on day 14 and 21 (Figure 6D). After EDS treatment, the bodyweights of rats were also decreased on the first 7 days. Subsequently, the bodyweights in ADSC‐LC transplanted rats or PBS injected rats started to recover, but both were still lower than that of control rats with PBS injection on day 14 and 21 (Figure 6E). These results suggested that ADSC‐LCs transplanted in rats had acquired some properties of LCs as they had the potential to restore the blood testosterone levels, and further recover the testis and bodyweights of EDS treated‐rats.

### 3.7 Enumeration of CYP11A1 positive Leydig cell number

All LCs could be identified by CYP11A1 staining because this enzyme was expressed in all LC lineages. Brown cytosolic staining in the testis interstitium shows CYP11A1 positive cells. At 14 or 21 days after exposure to EDS, CYP11A1 positive staining cells were observed in both groups, and the numbers of CYP11A1 positive staining cells in ADSC‐LC transplantation group were more than those of PBS injection group (Figure S1A). In addition, the quantification analysis showed that the cell number of ADSC‐LC transplantation group exceeding PBS injection group on day 14 was almost similar to that on day 21 (Fig. S1B). These data suggested that the increasing CYP11A1 positive cells might be mainly derived from the endogenous regenerated LCs, and ADSC‐LCs almost had no effects on the endogenous LCs.

## 4 DISCUSSION

Androgen deficiency is a very common physical disorder that not only affects adults but also jeopardizes children's health.^36^ Trauma, infections, tumour growth and radiation therapy can be the reasons for androgen deficiency.^37^ For children undergoing puberty who suffer from androgen deficiency, traditional androgen replacement therapy cannot mimic their natural testosterone fluctuations,^38^ and this may then lead to primary hypogonadism and altered sexual development.^39^ To avoid these side effects, paediatricians need to have alternative therapeutic options that simulate natural developmental fluctuations.^38^

As stem cells exhibit the potential to differentiate into multiple cell types, MSCs have been used widely in the treatment of organ dysfunction.^40^, ^41^, ^42^ Human adipose‐derived stem cells (ADSCs) are very similar to MSCs in terms of surface antigens and they also possess multipotentiality.^43^, ^44^ As adipose tissue can be more easily obtained than other tissues, ADSC is a more reliable stem cell source for therapy.^45^, ^46^ Therefore, in this study, we will try to induce human ADSCs differentiation into Leydig‐like cells (ADSC‐LCs), and transplanted these cells into the testes of EDS‐treated rats to investigate their therapeutic potential.

The developmental of LC lineages in vivo consists of four steps: stem LCs (undifferentiated mesenchymal‐like stem cells, SLCs), progenitor Leydig cells (PLCs), ILCs and adult Leydig cells (ALCs).^47^, ^48^, ^49^, ^50^ In our team previous review, we had systematically documented the effects of different factors such as leukaemia inhibitor factor (LIF), desert hedgehog (DHH), platelet‐derived growth factors (PDGF‐AA/BB), kit ligand (c‐kit), insulin‐like growth factor 1 (IGF1), transforming growth factor β (TGF‐β), activin, fibroblast growth factor 2 (FGF2), LH, androgen and others on the development (differentiation and proliferation) of LC lineages.^51^ Based on this review and practical experience, we optimized several factors to induce the differentiation of ADSCs into Leydig‐like cells in this study.

In the testis, DHH, secreted by Sertoli cells, is critical for LC development. Rat testis with a null mutation of DHH gene (*Dhh*) lacks ALCs.^52^ When added to cultured seminiferous tubules during 2 and 3 weeks, which LC differentiation occurs, SAG (an agonist of DHH) and Li resulted in at least 10‐fold stimulation of testosterone production.^27^ 22R‐hydroxycholesterol (22R‐OHC), a membrane‐permeant cholesterol analogue, can promote the enzymatic activity of CYP11A1 to synthetize steroid hormones of LCs.^53^ The combined utilization of SAG, 22R‐OHC and Li at the appropriate concentrations, could mimic the effect of transcription factor SF‐1 on the development of LCs. SF‐1 is an orphan nuclear receptor that belongs to the NR5A subfamily, which is essential for sexual differentiation and formation of the primary steroidogenic tissues.^54^ SF‐1 knockout mice completely lack adrenal glands and gonads and die soon after birth.^55^ During the first phase of differentiation from day 0 to 7, SAG, 22R‐OHC and Li were added into the ADSC‐DIM to induce the differentiation of ADSCs towards steroid‐like cells.

In addition, PDGF receptor a (PDGFRA) is expressed in the LC lineage cells. PDGF‐AA could not only significantly stimulate the proliferation of SLCs,^27^ but also stimulate the differentiation of stem LCs.^56^ FGF2 belongs to a heparin‐binding growth factor family. It can affect multiple cell functions, including proliferation, migration, survival and differentiation. FGF2 can dramatically stimulate LC proliferation.^27^ FGF2 can also stimulate immature LC steroidogenesis in the absence of LH, and has a biphasic effect on LH binding to its receptors (LHCGR) in ILCs, with low concentrations (0.1‐1.0 ng/mL) inhibitory and high concentrations (10‐100 ng/mL) stimulatory.^57^ Androgen receptor (NR3C4) is a nuclear receptor and is expressed in LC lineages and other testicular cells such as Sertoli cells and peritubular Myoid cells.^58^ Androgen profiles are still important to the development of LCs.^59^ During the second phase from day 7 to 14, PDGF‐AA, FGF2 and androgen were added into the ADSC‐DIM to further enhance the proliferation and differentiation of ADSCs.

LCs are the only cells that respond to LH in the testis.^60^ LH binds to the LHCGR in LCs, resulting in both acute‐ and trophic‐effects. Acute‐effects involve the mobilization and delivery of cholesterol to mitochondran to start the steroidogenesis. The trophic effects involve increases in gene transcriptions and steroidogenic enzyme activities. Both effects are required for the maintenance of an optimal steroidogenesis in the LCs.^60^ Without LH stimulation, LC steroidogenic enzyme activities are reduced. LH signalling is critical to both LC differentiation and proliferation.^61^ During the third phase from day 14 to 18, LH and SAG were added into the ADSC‐DIM to promote the maturity of differentiated ADSCs.

In this study, we demonstrated that ADSCs could be differentiated into Leydig‐like cells expressed cholesterol transporter: STAR and SCARB1 and steroidogenic enzymes: CYP11A1, HSD3B1, CYP17A1 and HSD17B3, and produced testosterone by few defined molecular compounds. Moreover, the result of flow cytometry showed that the HSD17B3 positive cells reached up to 34.74% in differentiated cells. Compared with other organs, testis is immunologically privileged.^62^ To investigate whether ADSC‐LCs have the ability to survive and function in the interstitium of rat testes in vivo, we transplanted ADSC‐LCs into an EDS‐treated rat, an androgen deficiency model, as previously described.^63^ EDS is an alkylating agent which has selective pro‐apoptotic effects on LCs.^31^ Approximately 2‐3 weeks after a single dose of EDS, newly regenerated LCs could be observed within the testicular interstitium.^64^ Approximately 8 to 10 weeks later, the LC population returned to its original size and had restored its ability to produce testosterone.^65^ Based on these results, we collected the testes of the cell‐transplanted rats on day 21 after EDS administration when the regenerated LCs appear a little to assess the state of the transplanted cells. As a result, we observed that the transplanted PKH26‐labelled ADSC‐LCs localized exclusively in the interstitium of the testis, and expressed LC‐specific marker CYP11A1. Importantly, these PKH26‐labelled ADSC‐LCs could survive at least 2 weeks in vivo, which demonstrated that they successfully integrated into the host niche. In addition, the blood testosterone levels of the ADSC‐LC transplanted rats remained higher than that of the EDS‐treated rats up to 21 days. As testosterone plays an important role in maintaining normal reproductive organs,^66^ testosterone deficiency can cause atrophy of reproductive organs.^4^ After exposure to EDS, the testis and bodyweights of EDS‐treated rats were dramatically decreased on day 7. Subsequently, however, these indicators in rats receiving ADSC‐LC transplantation could be restored faster than those of PBS injected rats with EDS treatment, but both were still lower than those of PBS injected rats without EDS treatment (control) on day 14 and 21.

In this study, we developed a novel differentiation protocol, to our knowledge, which is the first to demonstrate that ADSCs were able to be differentiated into testosterone‐producing Leydig‐like cells (ADSC‐LCs) by few defined molecular compounds but not bringing in the exogenous transcription factors. In addition, when ADSC‐LCs labelled with lipophilic red dye (PKH26) were transplanted into EDS treated rats, they could survive and function in the interstitium of testes, and accelerate the recovery of blood testosterone levels, testis weights and bodyweights. Our findings provide a new insight into the stem cell‐derived Leydig cell replacement therapies for the treatment of the patients with testosterone deficiency or decline.

## CONFLICT OF INTEREST

The authors have declared that no conflict of interest exists.
