# Supplementary figures and images for: Differentiation of human adipose derived stem cells into Leydig‐like cells with molecular compounds
Source: J Cell Mol Med. 2019 Jul 10;23(9):5956–69. doi: 10.1111/jcmm.14427 (PMC6714210; doi:10.1111/jcmm.14427)

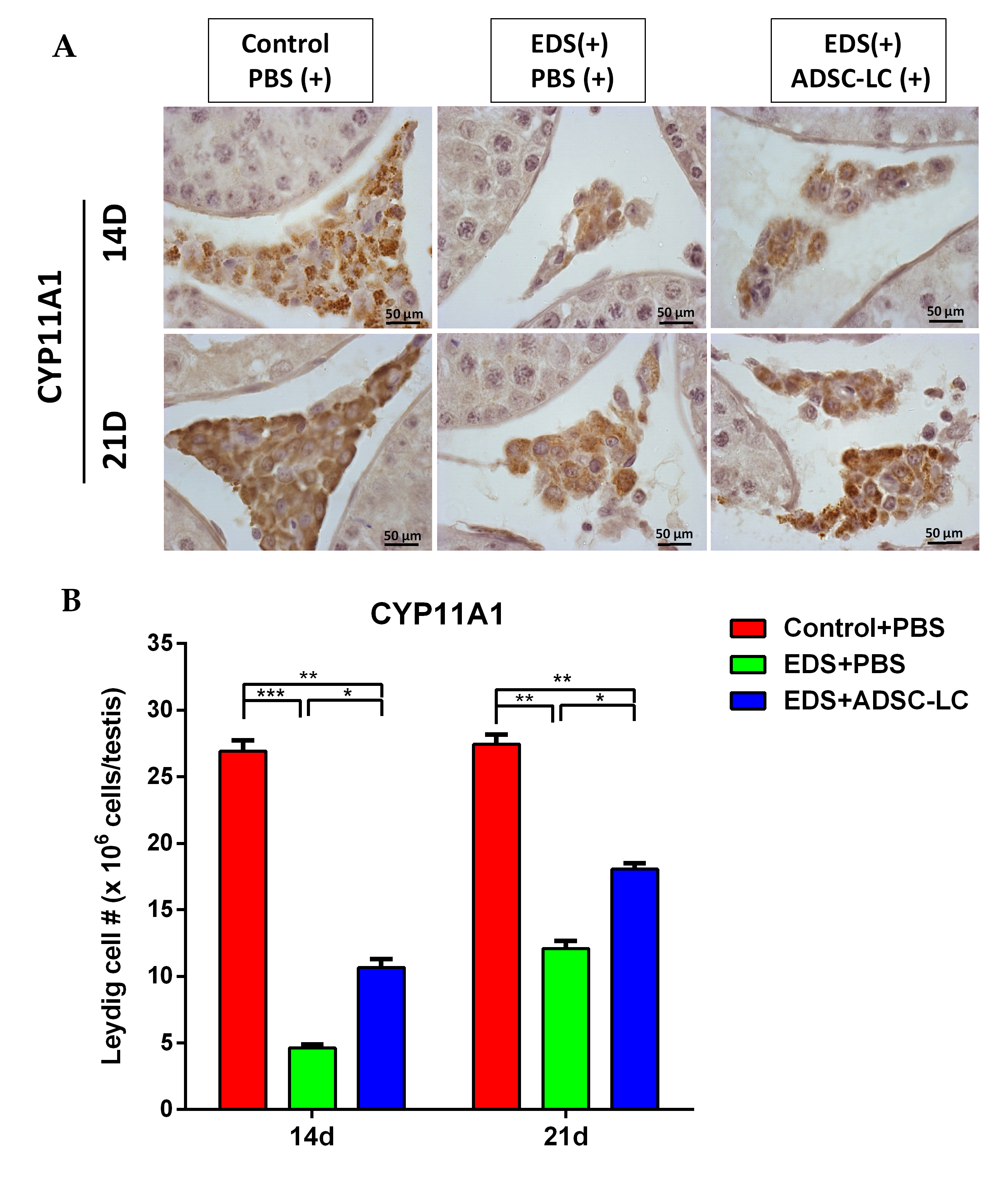

Supplement: Supplementary file 1 [file JCMM-23-5956-s001.tif]
